# Chemerin as an Inducer of β Cell Proliferation Mediates Mitochondrial Homeostasis and Promotes β Cell Mass Expansion

**DOI:** 10.3390/ijms24119136

**Published:** 2023-05-23

**Authors:** Min Li, Ruifan Zhang, Qian Ge, Lingzhi Yue, Dan Ma, Firas Khattab, Wenhua Xie, Yewei Cui, Patrick Gilon, Xueya Zhao, Xi Li, Rui Cheng

**Affiliations:** 1 Institute of Life Sciences, School of Basic Medicine, Chongqing Medical University, Chongqing 400016, China; 2020111815@stu.cqmu.edu.cn (M.L.); ruifanzhang@stu.cqmu.edu.cn (R.Z.); 2021110841@stu.cqmu.edu.cn (L.Y.); 2022110841@stu.cqmu.edu.cn (D.M.); 2020320292@stu.cqmu.edu.cn (W.X.); yeweicui299@stu.cqmu.edu.cn (Y.C.); hutuxueya@cqmu.edu.cn (X.Z.); 2 The First Clinical College, Chongqing Medical University, Chongqing 400016, China; geqianqian@aliyun.com; 3 Pôle d’Endocrinologie, Diabète et Nutrition, Institut de Recherche Expérimentale et Clinique, Université Catholique de Louvain, 1200 Brussels, Belgium; firas.khattab@uclouvain.be (F.K.); patrick.gilon@uclouvain.be (P.G.)

**Keywords:** chemerin, β cell, adipokine, insulin secretion, mitochondrial homeostasis, type 2 diabetes

## Abstract

Loss of the β cell population is a crucial feature of type 2 diabetes. Restoring the β cell mass by stimulating β cell proliferation and preventing its apoptosis was proposed as a therapeutic approach to treating diabetes. Therefore, researchers have been increasingly interested in identifying exogenous factors that can stimulate β cell proliferation in situ and in vitro. Adipokine chemerin, which is secreted from adipose tissue and the liver, has been identified as a chemokine that plays a critical role in the regulation of metabolism. In this study, we demonstrate that chemerin as a circulating adipokine promotes β cell proliferation in vivo and in vitro. Chemerin serum levels and the expression of the main receptors within islets are highly regulated under a variety of challenging conditions, including obesity and type 2 diabetes. As compared to their littermates, mice overexpressing chemerin had a larger islet area and increased β cell mass with both a normal and high-fat diet. Moreover, in chemerin-overexpressed mice, we observed improved mitochondrial homeostasis and increased insulin synthesis. In summary, our findings confirm the potential role of chemerin as an inducer of β cell proliferation, and they provide novel insights into the helpful strategy to expand β cell population.

## 1. Introduction

Obesity is a prevalent global health issue that increases the risk of developing type 2 diabetes mellitus (T2DM) [1], a condition characterized by hyperglycemia resulting from insulin resistance and pancreatic β-cell failure, leading to insufficient insulin secretion [2]. The primary approach to treating T2DM is to improve insulin sensitivity in peripheral metabolic tissues and enhance insulin secretion. However, in the process of T2DM, when the pancreatic islets begin to lose β-cell mass, no medication can correct the defective insulin secretion. The majority of patients with this condition must then rely on exogenous insulin therapy and become insulin-requiring. Restoring the loss of β-cell mass represents a significant challenge in treating this patient population [3]. Therefore, promoting pancreatic β-cell mass expansion is the most efficient therapeutic strategy to control β cell loss and prevent diabetes.

Adipose tissue, being the largest endocrine organ, plays a critical role in interorgan communication by secreting various adipokines, which act on other organs, including the liver, heart, muscle, and pancreas, to regulate metabolic homeostasis. The effect of adipokines on pancreatic islet β cells is, however, still conflicting. Some circulating adipokines, such as adiponectin, insulin-like growth factor 1 (IGF1), and visfatin, increase insulin synthesis and release, promote β cell proliferation [4]. Conversely, apelin and resistin inhibit insulin secretion, while retinol-binding protein 4 (RBP4) and leptin inhibit glucose-stimulated insulin secretion (GSIS) [4,5,6]. Asprosin can induce the apoptosis of MIN6 cells through the AMPK-mTOR pathway [7]. Among adipokines, chemerin has emerged as a significant predictor of obesity and T2DM, and it may mediate glucose-induced insulin secretion through its receptor [8].

Chemerin, a chemokine that recruits antigen-presenting cells, was first discovered in 1997 [9,10] and is encoded by the *RARRES2* gene with autocrine, paracrine, and endocrine functions [11]. Chemerin is primarily expressed in white adipose tissue and the liver, with small amounts also found in the muscles, kidneys, and lungs [12,13]. It exerts its biological and physiological function by binding to its receptors: chemokine-like receptor 1 (CMKLR1) [14], chemokine (CC motif) receptor-like 2 (CCRL2) [14,15,16], and G-protein coupled receptor 1 (GPR1) [16]. An increasing body of evidence from recently published studies suggests that chemerin plays an essential regulatory role in metabolism. It regulates adipocyte differentiation and controls beige fat thermogenesis through its receptor CMKLR1 [17]. Chemerin deficiency can enhance adipogenesis in subcutaneous adipose tissue [18] and in bone marrow. Moreover, chemerin promotes osteogenic differentiation and bone formation [19].

Chemerin has been extensively researched in cancer and inflammatory diseases. Its novel function was revealed in 2007 as a regulator of metabolic disorders such as obesity and T2DM [12]. The effect of chemerin on pancreatic β cells is not yet fully understood. Prior studies have yielded inconsistent findings. Specifically, serum chemerin levels have been found to be reduced in individuals with T2DM, while mouse islet β cells exhibit an abundant expression of chemerin, and the addition of chemerin in vitro has been shown to increase insulin secretion [8,20]. Conversely, Ferland et al. reported no chemerin expression in rodent pancreas. However, chemerin secretion by adipose tissue has been seen to improve insulin sensitivity in peripheral tissues [21]. The present study aims to elucidate the role of chemerin on β cells, including the mechanisms by which it regulates islet β cell mass and functions. Comprehensive research on how chemerin influences islet function and mass is crucial for understanding the pathogenesis of T2DM and identifying potential targets for T2DM treatment.

## 2. Results

### 2.1. The Circulating Chemerin Level Is Correlated with T2DM in Humans and Mice

**Figure 1:**
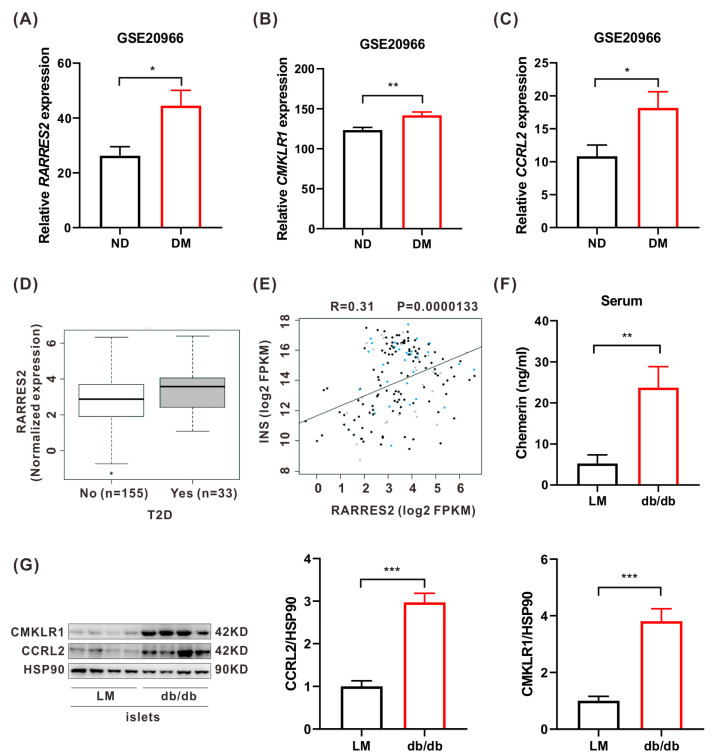
The levels of chemerin and its receptors: CMKLR1, and CCRL2 in T2DM humans and mice. (**A**–**C**) RNA-seq differential expression analysis of *RARRES2* (**A**), *CMKLR1* (**B**), and *CCRL2* (**C**) in human islets obtained from non-diabetic donors (n = 10) and T2DM donors (n = 10). (**D**) Expression of *RARRES2* in islets of non-diabetic (n = 155) and T2DM (n = 33) patients in the IGV database. (**E**) Correlation between *RARRES2* and *INS* in human islets. (black dots: normal glucose tolerance, NGT; cyan dots: impaired glucose tolerance, IGT; gray dots: T2DM). (**F**) ELISA detection of serum chemerin in LM and *db/db* mice (n = 6). (**G**) Western blot analysis of chemerin receptors’ expression relative to the endogenous control protein HSP90 in pancreatic islets of LM and *db/db* mice (n = 4). * *p* < 0.05; ** *p* < 0.01; *** *p* < 0.001. ND, non-diabetic; DM, diabetes mellitus; T2D, type 2 diabetes; LM, littermate.

Prior research has shown a positive correlation between serum chemerin levels and T2DM patients when compared to healthy controls [22,23]. To investigate the relationship between chemerin and T2DM, we analyzed the β cell gene expression profiles of 10 healthy individuals and 10 T2DM patients from the GSE20966 data set available in the GEO database. Our analysis identified three noteworthy genes, namely *Chemerin*, *Cmklr1*, and *Ccrl2*. The expression of chemerin and its receptors was significantly elevated in T2DM patient β cells (Figure 1A–C). This observation was further supported by the Islet Gene View database (https://mae.crc.med.lu.se/IsletGeneView/, accessed on 20 February 2023), which also reported an increased expression of *RARRES2* (encoding chemerin) in T2DM patients. Additionally, *RARRES2* expression was positively correlated with insulin gene expression (Figure 1D,E). We further validated these findings in the T2DM *db/db* mouse model (Figure S1A–C), which demonstrated that serum chemerin levels were higher in diabetic mice compared to their littermates (LM) (Figure 1F). These results validated the positive association between high circulating chemerin and T2DM. Moreover, we found that the protein expression levels of CMKLR1 and CCRL2 were significantly elevated in *db/db* mouse islets (*p* < 0.001), as determined by a Western blot (WB) analysis (Figure 1G).

### 2.2. The Expressions of Chemerin, CMKLR1, and CCRL2 in Islets and Peripheral Tissues

**Figure 2:**
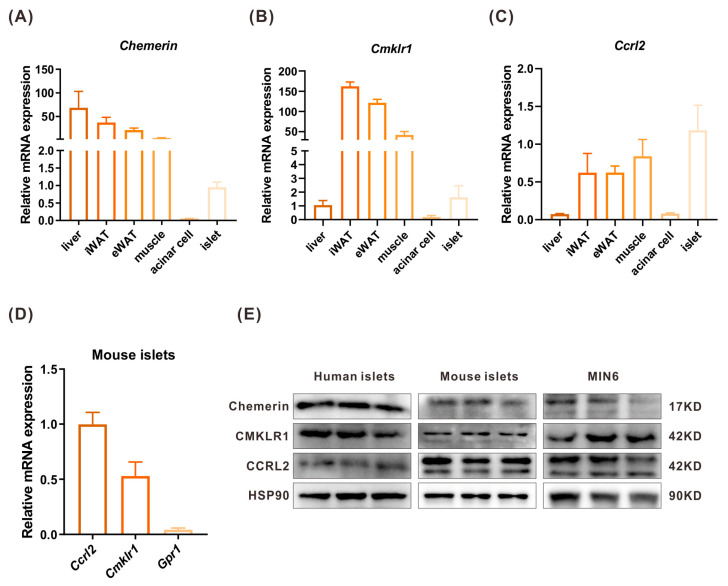
The expression of chemerin and its receptors CMKLR1 and CCRL2 in islets and peripheral tissues. (**A**–**C**) qPCR analysis of *Chemerin* (**A**), *Cmklr1* (**B**), and *Ccrl2* (**C**) expression in islets and peripheral tissues of mice (n = 3). (**D**) qPCR analysis of chemerin receptors’ expression in mouse islets (n = 3). (**E**) Western blot analysis of chemerin and its receptors expression in human islets, mouse islets, and MIN6 cells (n = 3).

Sixteen-week-old C57BL6 male mice were sacrificed to collect tissues such as the liver, inguinal white adipose tissue (iWAT), epididymis WAT (eWAT), muscle, acinar cell, and islet, from which RNA was extracted. The expressions of chemerin and its receptors CMKLR1 and CCRL2 were measured by WB and real-time quantitative PCR (RT-qPCR) in pancreatic islets and other peripheral tissues. The RT-qPCR analysis showed that chemerin was expressed at a high level in the adipose and the liver, a moderate level in muscle, and a lower level in the islet. In contrast, there was no expression in the acinar cell (Figure 2A). *Cmklr1* was highly expressed in adipose tissue and muscle, whereas the expression levels in the liver and islet were much lower (Figure 2B). The *Ccrl2* expression level was higher in the islets than in other peripheral tissues (Figure 2C) and it was the main chemerin receptor in mouse islets (Figure 2D). Chemerin, CMKLR1, and CCRL2 protein levels were measured by WB, and the results showed that chemerin and its receptors were expressed in human and mouse islets and MIN6 cells. Moreover, chemerin’s expression level in human islets was higher than in mouse islets and MIN6 cells (Figure 2E).

### 2.3. Chemerin Can Promote the Proliferation and Inhibit the Apoptosis of MIN6 In Vitro

**Figure 3:**
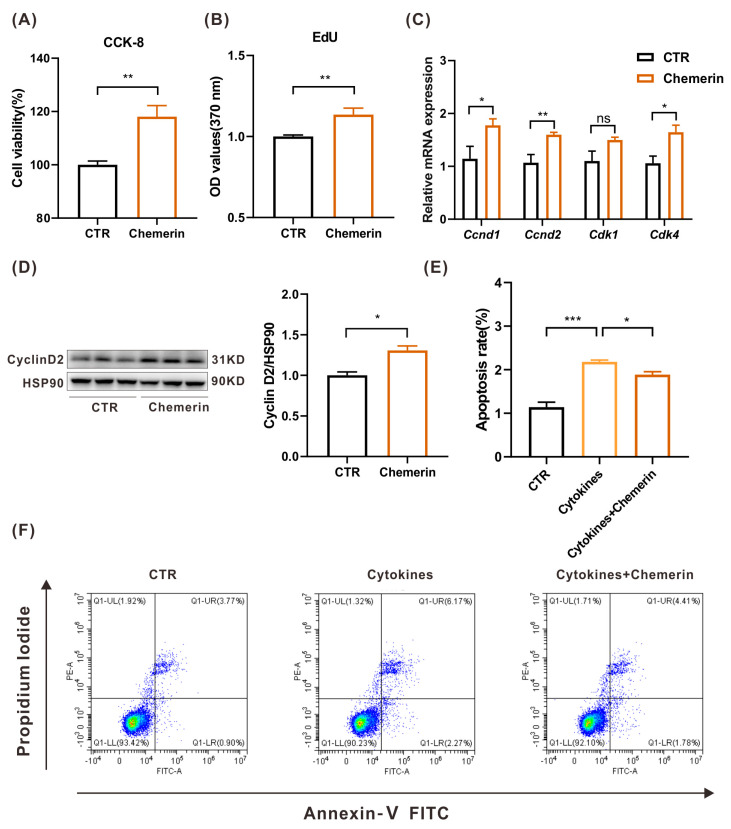
Chemerin can promote the proliferation and inhibit the apoptosis of MIN6 cells in vitro. (**A**) Cell viability was determined by CCK−8 assay in MIN6 cells treated with chemerin (n = 6). (**B**) MIN6 cells proliferation detected by EdU assay (n = 6). (**C**) qPCR analysis of cell cycle-related gene expression in MIN6 cells treated with chemerin (n = 6). (**D**) The protein expression level of cell proliferation-related protein Cyclin D2 was evaluated by Western blot (n = 3). (**E**,**F**) Analysis of apoptosis level in MIN6 cells treated with chemerin in the presence of cytokines (IFNγ, 10 ng/mL; IL-1β, 10 ng/mL; TNFα, 10 ng/mL) stimulation analyzed by flow cytometry (n = 4). Results are shown by color density plots, each dot represents a single cell, and the cell number is represented by different colors in a given region (red: high cell density; green: modest cell density; blue: low cell density). * *p* < 0.05; ** *p* < 0.01; *** *p* < 0.001; ns, not significant.

It is widely acknowledged that islet β cell failure is the most significant factor in the development of T2DM. β cell loss causes insufficient insulin secretion and further hyperglycemia in the body. We assayed β cell death and proliferation to assess whether chemerin plays a role in preserving β cell mass. We observed that MIN6 cell viability significantly increased after chemerin treatment using the Cell Counting Kit-8 test (Figure 3A). A 5-ethynyl-2′-deoxyuridine (EdU)-based assay showed that chemerin promoted MIN6 cell proliferation in vitro (Figure 3B). Therefore, we detected the expression of cell cycle-related genes Cyclin D1 (*Ccnd1*), Cyclin D2 (*Ccnd2*), Cyclin-Dependent Kinase 1(*Cdk1*), and 4 (*Cdk4*). The RT-qPCR result revealed a remarkable increase in the expression level of those genes in chemerin-treated cells (Figure 3C), suggesting that chemerin plays a vital role in cell growth. Among the above genes, the cell cycle protein Cyclin D2 is known to be a critical factor that controls cell proliferation in islet β cells [24,25]. Thus, we detected the expression of Cyclin D2 and found that the Cyclin D2 level was indeed increased after chemerin treatment in MIN6 cells (Figure 3D). These results revealed that exogenous chemerin could promote the proliferation of β cells by activating Cyclin D2.

Then, the apoptosis of MIN6 cells was measured by Annexin V-FITC. Annexin V is an intercellular protein that can bind to phosphatidylserine (PS). In a healthy cell, PS is located on the inner leaflet of the plasma membrane. While in an apoptosis cell, PS is translocated onto the outer leaflet of the plasma membrane; Annexin V-FITC can thus bind to PS and specifically target the apoptotic cell. Our results showed that with chemerin, the apoptosis of MIN6 cells was downregulated under normal conditions (Figure S2A). In the more challenging circumstance of cytokine treatment (IFNγ, IL-1β, TNFα; 10 ng/mL) induced cell death, adding chemerin significantly decreased MIN6 cell apoptosis (Figure 3E,F). The above results provide compelling evidence that chemerin exerts a protective effect on β cell mass by promoting cell proliferation and inhibiting cell death.

### 2.4. Islets Area and β Cell Mass Are Upregulated in Chemerin-Overexpressed Transgenic Mice

**Figure 4:**
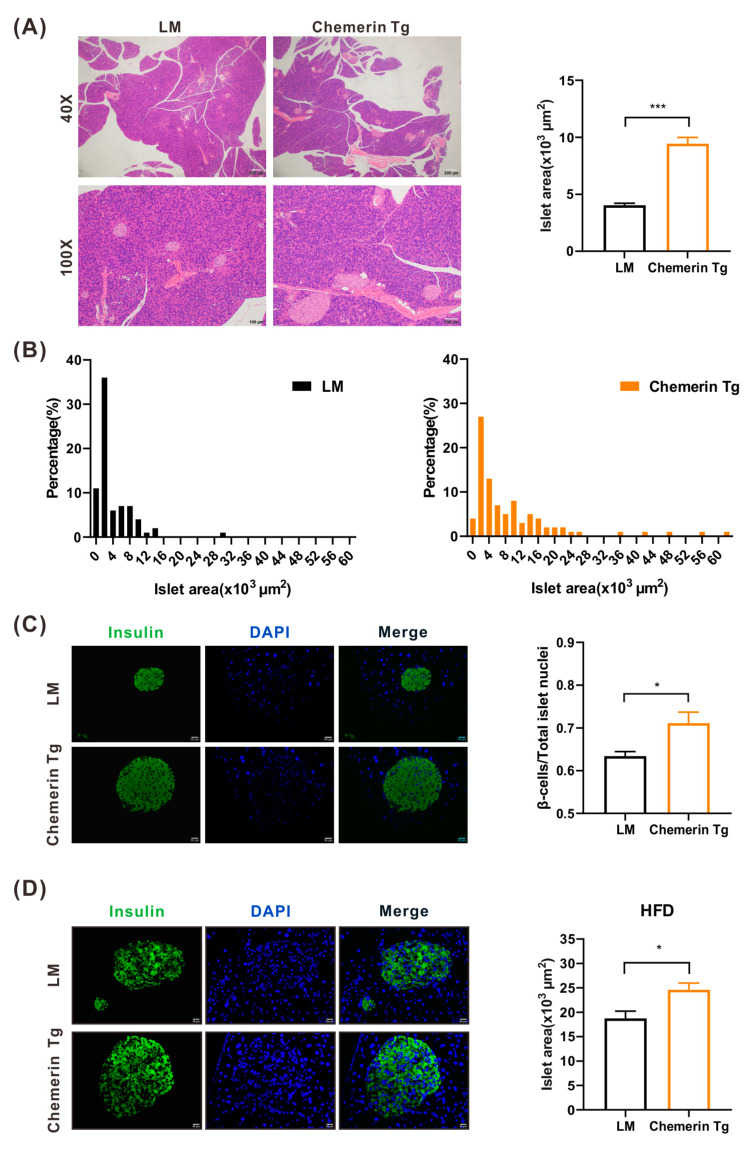
Islets area and β cell mass were upregulated in chemerin transgenic mice. (**A**) H&E staining of pancreas tissues from LM and chemerin-Tg mice (n = 3). (**B**) Islets area distribution in LM and chemerin-Tg mice. (**C**) Immunofluorescence staining of pancreas tissues from LM and chemerin-Tg mice (n = 3). (**D**) Immunofluorescence staining of pancreas tissues from LM and chemerin-Tg mice fed with HFD (n = 3). * *p* < 0.05; *** *p* < 0.001; LM, littermate; Tg, transgenic; HFD, high-fat diet.

To further investigate whether chemerin has a positive effect on improving β cell mass in vivo, *Ap2* (adiponectin) drive chemerin-overexpressed transgenic mice (chemerin-Tg mice) were raised and utilized as described previously [19] (Figure S3A–C). H&E staining of pancreatic islets and immunofluorescence (IF) staining of β cells were performed in twenty-week-old male chemerin-Tg mice with a normal or high-fat diet, and then compared to their littermates’ (LM) mice. It was evident that in chemerin-Tg mice, the size of the islets was greater than in LM mice (Figure 4A). Statistical analysis showed that the area of the islets in mice overexpressing chemerin became larger. The percentage of islets of different sizes was counted, and it was observed that the proportion of large islets was mainly increased in chemerin-Tg mice (Figure 4B). Moreover, the IF staining of β cells also demonstrated that the ratio of β cells amount/total islet cells was higher in the islets of chemerin-Tg mice than it was in the LM mice islets (Figure 4C). At HFD, β cells may expand to compensate for high blood glucose by increasing insulin secretion and replenishing β cell mass. Surprisingly, we discovered that after high-fat feeding, the size of the islets from chemerin-Tg mice was more considerable than the islets from LM (Figure 4D). It is well documented that pancreatic β cells have a normal lifespan. In normal physiological conditions, the replication capacity of β cells drops with age after weaning; they rarely replicate during a normal lifetime [26,27], and mature β cells do not respond to different proliferation stimuli [28]. Our results revealed that pancreatic β cell mass augments when overexpressing chemerin under normal physiological and obese conditions; this could be a potential direction for T2DM treatment.

### 2.5. Chemerin Sustains Mitochondrial Homeostasis to Prevent Apoptosis

**Figure 5:**
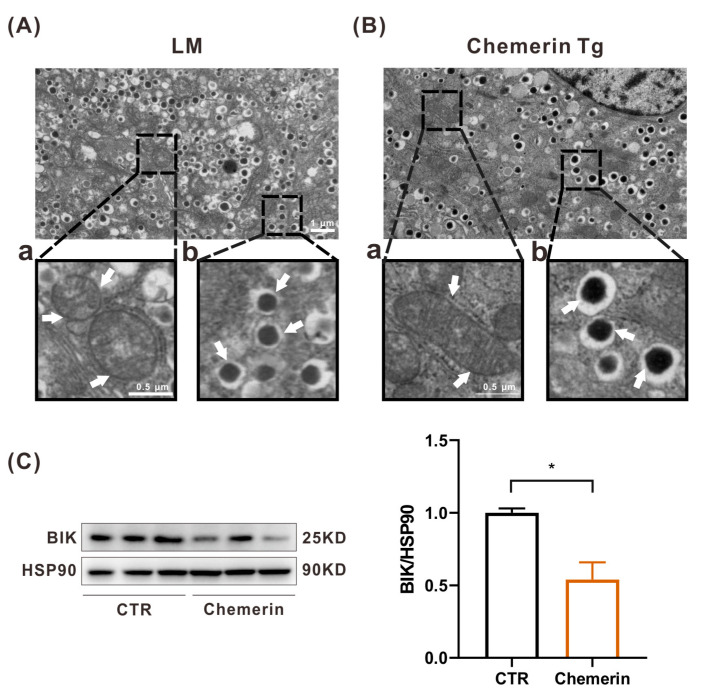
Chemerin sustains mitochondrial homeostasis to prevent apoptosis. (**A**,**B**) Transmission electron microscope images of pancreas tissues from LM and chemerin-Tg mice: mitochondria (**a**) and insulin secretory granules (**b**). (**C**) The protein level of BIK relative to the endogenous control protein HSP90 was evaluated by Western blot (n = 3). * *p* < 0.05.

There are two main pathways of apoptosis: the intrinsic or mitochondrial pathway and the extrinsic or death receptor pathway [29,30]. Since we observed that exogenous chemerin prevented MIN6 cell apoptosis in normal conditions, we investigated whether chemerin exerts a regulatory effect on mitochondria in vivo. Mitochondria are called the “powerhouse” within cells. Their membrane-bound structure is essential to produce energy in the form of ATP and to keep cells alive. Furthermore, mitochondria can regulate cell signaling and heat generation, and they are also the most crucial organelle controlling cell growth and apoptosis. The pancreas was collected from LM and chemerin-Tg mice; after slide preparation, β cells were observed using the JEM-1400-FLASH transmission electron microscope. Although most of the mitochondria in the β cells of LM mice showed bean-like or oval-shaped and well-placed widthwise inner membranes, small amounts of mitochondria had an irregular cristae distribution and empty vacuoles in the center (Figure 5Aa). We observed that in the β cells of chemerin-Tg mice, the mitochondria were mostly bean-shaped with a similar size, the internal structure was compact, and the inner membranes were vertical to the mitochondrion axis (Figure 5Ba). BCL2 interacting killer (BIK) protein initiates apoptosis by reorganizing mitochondrial cristae [31]. At this point, we detected the BIK protein level in MIN6 cells. The WB result showed that BIK expression was markedly reduced when adding exogenous chemerin in a cell culture medium for 24 h (Figure 5C). These findings indicated that chemerin prevents the apoptosis of β cells by maintaining mitochondrial homeostasis.

### 2.6. Chemerin Improves β Cell Function In Vitro

**Figure 6:**
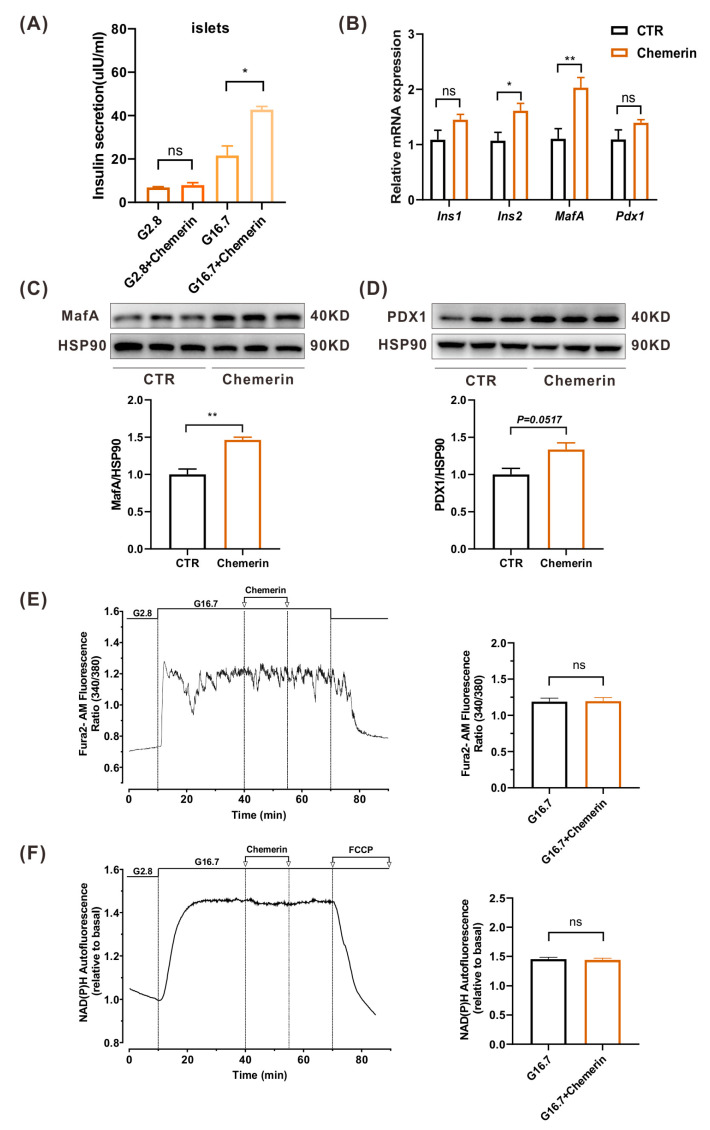
Chemerin promotes β cell function. (**A**) Effects of chemerin on insulin secretion of mice at different glucose concentrations. (**B**) qPCR analysis of *Ins1*, *Ins2*, *MafA*, and *Pdx1* genes’ expression in MIN6 cells treated with chemerin (n = 6). (**C**,**D**) The β cell function-related proteins’ MafA (**C**) and PDX1 (**D**) expressions were detected by Western blot (n = 3). (**E**,**F**) Effect of chemerin on the [Ca^2+^]_c_ (**E**) (n = 8 islets/3 mice) and NAD(P)H autofluorescence (**F**) (n = 4 islets/3 mice) of mice islets with 16.7 mM of glucose. Chemerin (200 ng/mL) and FCCP (10 µM) were added as indicated. * *p* < 0.05; ** *p* < 0.01; ns, not significant. FCCP, an uncoupler of mitochondrial oxidative phosphorylation.

The dense core of insulin secretory granules comprises pro-insulin, Zinc, insulin hexamer, membrane proteins, co-secretory factors, enzymes, etc. [32]. The dense core of zinc–insulin hexamer crystals is formed in the mature insulin granules. The darker the color, the higher the content of the zinc–insulin crystal [33]. Interestingly, we found that the insulin secretory granules were larger and had higher density in chemerin-Tg mice than in LM mice (Figure 5Ab,Bb). Thus, our finding implied that chemerin could regulate β cell function. Next, the GSIS was measured using primary isolated islets from wild-type C57BL/6J mice to verify whether chemerin could increase insulin secretion and regulate β cell function. At a low glucose concentration (2.8 mM), chemerin does not affect insulin secretion, whereas at a high glucose concentration (16.7 mM), it strongly increases insulin release (Figure 6A); this result is supported by the increased gene expression of *Ins1* and *Ins2* (Figure 6B). Moreover, by testing the expression level of MafA and PDX1, two crucial transcription factors that control pancreatic β cell’s identity, maturation, and function, we found that adding chemerin enhanced their expression in MIN6 cells with *p* = 0.0047 and *p* = 0.0517, respectively (Figure 6C,D). GSIS involves a triggering pathway and an amplifying pathway [34]. In both pathways, glucose metabolism plays an essential role. An increase in the ATP-to-ADP ratio closes K_ATP_ channels, and subsequently the influx of Ca^2+^, which triggers the exocytosis of insulin secretory granules [34]. Concurrently, glucose metabolism enhances the secretory response by increasing the efficiency of Ca^2+^. NAD(P)H fluorescence can be an indicator of glucose metabolism change [35]. In order to investigate the cause of the stimulation of insulin release induced by chemerin (Figure 6A), the cytosolic Ca^2+^ concentration [Ca^2+^]_c_ and NAD(P)H fluorescence were measured. The addition of 16.7 mM of glucose significantly increased [Ca^2+^]_c_ and NAD(P)H fluorescence. However, the addition of chemerin did not affect both parameters (Figure 6E,F and Figure S4C,D). There was no effect of chemerin at 2.8 mM of glucose on both [Ca^2+^]_c_ and NAD(P)H fluorescence (Figure S4A,B,E,F). The insulinotropic effect of chemerin does not result from a stimulation of the triggering or the amplifying pathway of glucose in β cells.

### 2.7. Chemerin Improves Glucose Tolerance In Vivo

**Figure 7:**
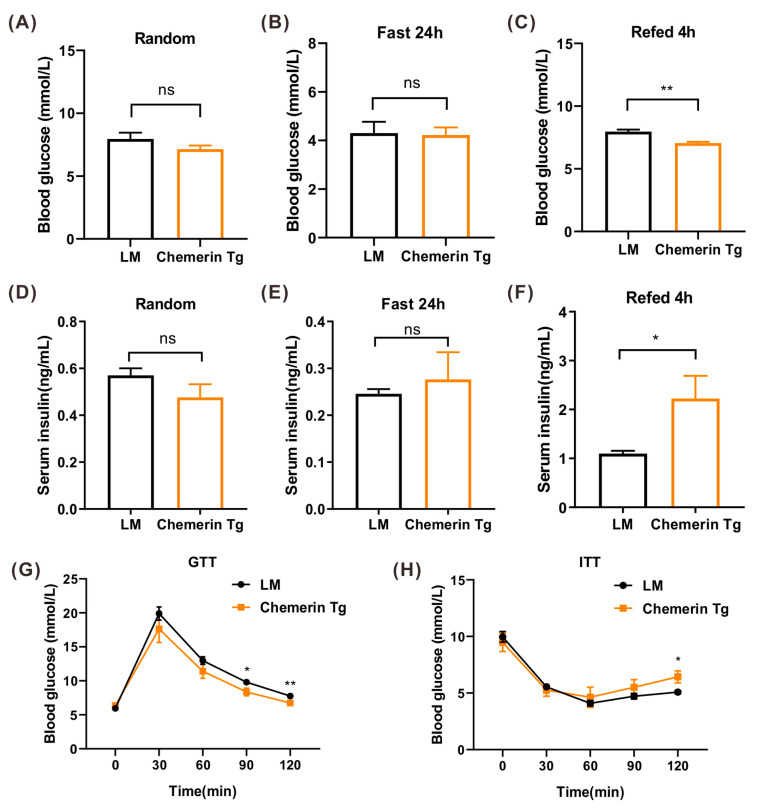
The ability of blood glucose regulation was increased in chemerin Tg mice. (**A**–**C**) Random (**A**), fasting (**B**), and refed 4 h (**C**) blood glucose in LM and chemerin Tg mice (n = 4). (**D**–**F**) ELISA analysis of random (**D**), fasting (**E**), and refed 4 h (**F**) serum insulin in LM and chemerin Tg mice (n = 4). (**G**,**H**) GTT and ITT assay in LM and chemerin Tg mice (n = 4). * *p* < 0.05; ** *p* < 0.01; ns, not significant.

In vivo study showed no difference between LM and chemerin-Tg mice in random glycemia (Figure 7A). After fasting for 24 h, the mice were refed. Glycemia was measured after 24 h of fasting and then again 4 h after refeeding. There was no difference at fasting, but blood glucose fell significantly 4 h after refeeding (Figure 7B,C). The serum insulin concentration was tested simultaneously, and there were no differences in random and fasting serum insulin concentration (Figure 7D,E), whereas a significant increase was observed at 4 h after refeeding (Figure 7F). This observation may explain the decreased blood glucose concentration 4 h after refeeding.

After GTT and ITT were performed, we noticed that LM mice and chemerin-Tg mice both showed excellent glucose tolerance (Figure 7G); however, in Chemerin-Tg mice, the blood glucose concentration was inferior to LM mice during the actual test, with a significant reduction at the time points of 90 min and again at 120 min (Figure 7G). IPITT showed that chemerin-Tg mice had similar insulin sensitivity to LM mice (Figure 7H). Despite the observed increase in the islet area and proportion of β cells in chemerin-Tg mice, there was no evidence of hyperinsulinemia.

The above findings corroborate that chemerin prevents β cell apoptosis by maintaining mitochondrial homeostasis. Furthermore, under physiological conditions, it can positively regulate β cell mass and function, enhance GSIS without affecting insulin sensitivity, and consequently better control blood glucose in vivo.

## 3. Discussion

The progressive loss of the mass and function of islet β cells plays an important role in the pathogenesis of T2DM, ultimately resulting in β cell death. Currently, there is no clinical treatment available for β cell failure and death. Our research provides hitherto undocumented evidence that the adipokine chemerin can directly impact pancreatic β cells. It can protect β cells from apoptosis and promote β cell proliferation and function. In chemerin-overexpressed mice, we observed an increase in the islet area and β cell mass, improving glucose tolerance. Our findings suggest that adipokine chemerin has the potential to regulate glucose homeostasis.

As a vital metabolic organ, adipose tissue secretes a generous amount of adipokines. These adipokines flow into the bloodstream; exert autocrine, paracrine, and endocrine functions; crosstalk with various organs; and influence whole-body metabolism. Communication between adipose tissue and pancreatic β cells is two-way and essential to maintaining β cell mass and wellness. The present study sought to solve the problem of β cell loss by focusing on the challenge of how to help β cells to survive and thrive. Several adipokines have been documented to affect β cell proliferation and apoptosis [4]. Leptin was the first adipokine characterized and discovered to be involved in directly regulating pancreatic β cells [36]. Leptin can prevent β cell death by increasing free fatty acid (FFA) oxidation [37]. In Zucker diabetic fatty (ZDF) rats, the deficiency of the leptin receptor promoted β cell death, indicating the direct effects of leptin via its receptor on β cells [38]. Furthermore, leptin has been reported to enhance β cell proliferation by activating the MAPK and JAK/STAT signaling pathways in MIN6 cells [39]. In addition, leptin was involved in PTEN inhibition, thereby upregulating β cell proliferation through activating cyclin-dependent kinase in *db/db* mice [40]. Adiponectin is another well-known adipose tissue-specific adipokine [41]. It can not only increase insulin secretion [42,43,44] and content [45], but also upregulate MIN6 cell proliferation via PPARγ-dependent mechanisms [45], increase β cell viability, and suppress β cell apoptosis by activating ERK and AKT in MIN6 cells and mouse islets [44]. Adiponectin can also upregulate insulin secretion and cell viability in rat β cell line BRIN-BD11 through ERK1/2 activation [46]. Visfatin is another adipokine implicated in β cell apoptosis. It can bind to insulin receptors, protect against palmitate-induced islet β cell apoptosis, and stimulate MIN6 cell proliferation [47]. The above information focuses on the positive effect of adipokines involved in β cell proliferation with respect to eventual treatment to rescue β cell loss. In the present study, we provide evidence that chemerin can preserve β cell survival and sustain mitochondrial homeostasis in normal conditions, suggesting that chemerin plays an important role in β cell mass and glycemia control.

The effect of chemerin on pancreatic β cells has received minimal study. The only available research was conducted by Takahashi et al. [15], who demonstrated that chemerin and its receptor CMKLR1 (also called ChemR23) are expressed in mouse islets. Chemerin is necessary for maintaining glycemia in vivo. There was no difference in net insulin sensitivity between chemerin-deficient mice and liver-overexpressed chemerin-Tg mice. The IPGTT result showed a significant decrease in glucose concentration in chemerin-Tg mice compared with chemerin knock-out mice, indicating that circulating chemerin in vivo could enhance insulin secretion via CMKLR1. In our study, the unaffected glucose metabolism was displayed by no change of intracellular Ca^2+^ and NAD(P)H concentrations after adding chemerin at 16.7 mM of glucose, revealing that chemerin increases insulin secretion in incubation experiments by boosting insulin synthesis and crystallization, as shown in Figure 5Bb and Figure 6B. Our IPGTT and IPITT results showed that chemerin-Tg mice have better glucose tolerance, and higher serum chemerin did not affect insulin sensitivity, which aligns with their findings. However, we noticed that in mouse islets, chemerin’s expression is weak compared to human islets. The expression of receptor CCRL2 is more prominent in mouse islets. Since it was established that CCRL2 has no downstream signaling pathway after binding to chemerin but served as a recruiter of circulating chemerin and presented them to CMKLR1 [48], we deduced that it was exogenous but not intrinsic chemerin that plays a vital role on islets. 

Takahashi et al. also showed chemerin and CMKLR1 mRNA levels in the pancreas collected from HFD and *db/db* mice. They first proposed that CMKLR1 expression level did not change when mice were fed with an HFD or in *db/db* mice. Secondly, the expression of chemerin was similar in HFD mice versus control mice. However, there was a remarkable reduction in *db/db* mice. Our study demonstrated that CMKLR1 and CCRL2 were intensely expressed in *db/db* islets compared to LM, which is consistent with the GEO database (GSE20966) showing that chemerin and its receptors’ expression are positively related to T2DM (Figure 1A–C).

Additionally, a previous study from our group reported that serum chemerin levels were upregulated in HFD-fed mice [19]. We found a similar result in *db/db* mice, suggesting that the serum chemerin concentration is related to adipose tissue expansion. The high level of circulating chemerin in obese conditions may reveal a chemerin resistance like high insulin blood concentration. It remains elusive whether this increased serum chemerin level plays an important role in balancing glycemia in metabolic disorder conditions.

Notably, compared with their littermates, mice overexpressing chemerin had a larger islet area and increased β cell mass with both normal and high-fat diets, suggesting that chemerin may exert an additional effect on β cell compensation. However, the intrinsic relationship among the different molecular mechanisms remains to be deeply investigated.

To summarize, our results cast a novel light concerning the direct effect of adipokine chemerin on pancreatic islets and glucose homeostasis. The present study proves that chemerin can positively regulate β cell function and prevent β cell apoptosis by maintaining mitochondrial homeostasis. Most importantly, chemerin could be a potent therapeutic regulator in expanding β cell mass by promoting their proliferation to treat T2DM. Our conclusions in this paper provided experimental data and theory support; nevertheless, further investigations are necessary to elucidate the exact effect of chemerin on obesity and T2DM.

## 4. Materials and Methods

### 4.1. Animal Models

Wild-type C57BL/6J mice, eight-week-old male C57BLKS/J *db/db* mice, and their control littermates were purchased from GemPharmatech (Nanjing, China). Transgenic (Tg) mice with *Rarres2* overexpression were generated as described previously [19]. A 12 h cycle of light/dark was used to house the mice in standard cages. Standard diet and water were available ad libitum. During the high-fat diet (HFD) feeding experiments, chemerin- Tg mice and their littermates were fed the diet (Research Diet, D12492, 60% kcal fat) for 14 weeks. All animal experiments were approved by Institutional Animal Care and Use of Chongqing Medical University and followed the Guidelines for Animal Experiments.

### 4.2. Glucose and Insulin Tolerance Test

Mice were fasted overnight for 14 h before the glucose tolerance test (GTT). The next day, we measured the weight and fasting blood glucose (FBG) of the mice and intraperitoneally injected them with a 25% glucose solution (1 mg/g). After that, the blood glucose was measured at 30, 60, 90, and 120 min. For the insulin tolerance test (ITT), we fasted the mice for 4 h; then, we measured their weight and fasting blood glucose (FBG). An intraperitoneal injection of insulin (0.75 mU/g) was administered to the mice. Next, the blood glucose was measured at 30, 60, 90, and 120 min.

### 4.3. Pancreatic Islets Isolation

Pancreatic islets from mice were digested by collagenase P (Roche, 0.65 mg/mL, Basel, Switzerland) for 15 min, separated with histopaque-1077 (Sigma, 10771, Saint Louis, MO, USA) density gradient centrifugation, and hand-picked with a stereomicroscope. We cultured the islets in RPMI-1640 medium, which was supplemented with 5 g/L of BSA (Sigma, A1933) and 1% penicillin/streptomycin (P/S) (Beyotime, C0222, Shanghai, China), at 37 °C, 5% CO_2_, for the next experiments.

Human pancreatic tissues were collected from the First Affiliated Hospital of Chongqing Medical University from non-diabetic patients undergoing partial pancreatectomy for chronic pancreatitis and benign pancreatic tumors. After resecting, the pancreatic tissue was placed in a chilled vessel with Belzer UW cold storage solution (Preservation Solutions, CHD081622, Wisconsin, USA) and taken to the laboratory for immediate processing. The digestive enzyme solution (130 mL of RPMI 1640 with 20 mL of 5 mg/mL Liberase DL (Roche, 54435300) was injected into the pancreatic tissue. The sample was cut into 2–3 mm^3^ pieces and shaken by hand in a 37 °C water bath for 10–15 min. Following digestion, cold RPMI 1640 medium was used to wash the digested pellets three times. The free islets were hand-picked using a dissecting microscope and cultured in RPMI 1640 medium supplemented with 10% FBS and 1% P/S.

### 4.4. Glucose-Stimulated Insulin Secretion (GSIS)

Isolated mouse islets were separated into a 24-well plate (3 islets/well) and preincubated for 1 h at 37 °C in 500 μL of KRBS buffer containing 2.7 mM of glucose. Then, the islets were incubated in 500 μL of KRBS buffer containing 2.7 mM or 16.7 mM of glucose for 1 h, simultaneously treated with or without chemerin (200 ng/mL) (Peprotech, 300-66, Chicago, IL, USA). Supernatants were collected after incubation for the measurement of insulin using an ELISA Kit (ThermoFisher, EMINS, Waltham, MA, USA).

### 4.5. H&E Staining and Immunofluorescence Staining

Paraformaldehyde (4%) was used to fix the pancreas samples, after which they were embedded in paraffin and cut into sections of 7 μm thickness. Following standard procedures, H&E staining and immunofluorescence staining were carried out. Then, we observed the section by a fluorescence microscope (Olympus, Shinjuku, Tokyo, Japan). For each sample, Image J software was used to perform a quantitative histomorphometric analysis of islet area and number. All islet areas in the sections from three individual mouse samples were calculated in each group using Image J software; then, the average islet area was calculated for each mouse. The Student’s t-test was used to compare the mean islet area between the two groups, and a frequency distribution test was conducted for the islet area of distribution percentage. The threshold of statistical significance was set at 0.05. Antibodies are shown in Table S1.

### 4.6. Cell Culture

The MIN6 cells (passage 5–25; mycoplasma negative) (kindly provided by Endocrinology, Diabetes and Nutrition Unit, University of Louvain, Ottignies-Louvain-la-Neuve, Belgium) were cultured in DMEM supplemented with 10% fetal bovine serum (FBS) (Gibco, A3161002C, Rockville, MD, USA), 10 mM of HEPES (Sigma, H0887, Saint Louis, MI, USA), and 1% P/S at 37 °C, 5% CO_2_.

### 4.7. Apoptosis Assay

To investigate the effects of chemerin treatment on MIN6 cell apoptosis, we used Annexin V-FITC/PI staining (Absin, abs50001, Shanghai, China). In 12-well plates, the cells were seeded at a density of 1 × 10^5^ cells per well. Then, the cells were treated with chemerin (200 ng/mL) for 24 h. Following the standard protocol, 300 μL of 1× Binding Buffer was used to resuspension the cells. Then, each cell suspension was gently mixed with Annexin V-FITC and PI. Following incubation for 15 min in the dark, the samples were measured by CytoFLEX (Beckman coulter, CytoFLEX, Brea, CA, USA).

### 4.8. EdU Cell Proliferation Assay

We performed a proliferation assay using an EdU Kit (Beyotime, C0088S, Shanghai, China). In a 96-well plate, cells were seeded at a density of 2 × 10^4^ cells per well and treated with chemerin (200 ng/mL) for 24 h. We followed the manufacturer’s protocol for the other steps. In the end, we used a microplate reader to measure absorbance at 370 nm.

### 4.9. Cell Counting Kit-8(CCK-8) Cell Viability Assay

The MIN6 cells (2 × 10^4^ cells/well) were seeded in a 96-well plate and treated with chemerin at the indicated concentration for 24 h. Next, each well was added to 10 μL of CCK-8 solution (TargetMol, C0005, Boston, MA, USA) and cultured for 2 h at 37 °C. In the end, we used a microplate reader to measure absorbance at 450 nm.

### 4.10. Western Blot

Protease and phosphatase inhibitor-containing lysis solution was used to lyse cells on ice for 30 min. We centrifugated the lysates to collect the supernatants, then quantitated and degenerated the supernatants. By using SDS-PAGE, equal amounts (20 μg) of protein lysates were separated, and they were electrophoretically transferred to PVDF membranes. A 5% skimmed milk block was applied to the membranes, followed by incubation with diluted antibodies. Secondary antibodies were incubated for 1 h on the membranes the next day. All antibodies are shown in Table S1.

### 4.11. Real-Time Quantitative PCR

According to the manufacturer’s protocol, total RNA was isolated by TRIzol^TM^ reagent (ThermoFisher, 15596026, Waltham, MA, USA) and reverse transcribed to cDNA with reverse transcriptase (ThermoFisher, 00698284, Waltham, MA, USA). SYBR Green (ThermoFisher, 00736756, Waltham, MA, USA) was used to measure the gene expression levels. *18S* and *Ppia* were used for normalization. We calculated the data using a 2^−ΔΔCT^ method. All primers were synthesized by Beijing Tsingke Biotech Co., Ltd. (Beijing, China). (shown in Table 1).

### 4.12. Transmission Electron Microscope

The pancreas was prefixed with 3% glutaraldehyde, followed by 1% osmium tetroxide post-fixation, series acetone dehydration, extended Epox 812 infiltration, and embedding. We used a diamond knife to slice ultrathin sections, then stained sections with methylene blue for orientation and stained sections with lead citrate and uranyl acetate. Observations of the sections were performed using the JEM-1400-FLASH transmission electron microscope.

### 4.13. [Ca^2+^]_c_ and NAD(P)H Fluorescence Measurements

Islets were placed in a 1.5 mL chamber and perifused at 37 °C at a flow rate of 0.5 mL/min. The chamber was mounted on a Zeiss Axiovert 100 inverted microscope equipped with a 40× objective. A sCMOS Prime 95 B (Photometrics, Tucson, AZ, USA) camera controlled by Metafluor software was used to acquire the images. Fura-2 AM (ION Biosciences, San Marcos, TX, USA) was loaded into the islets at 37 °C for 75 min in the culture medium. [Ca^2+^]_c_ and NAD(P)H fluorescences were detected as previously described [35,49].

### 4.14. Statistical Analysis

All data were presented as means ± SEM and analyzed in GraphPad prism 8. The unpaired two-tailed Student’s *t*-test was used to analyze the comparisons between the two groups. A *p* of < 0.05 was considered statistically significant.

## Figures and Tables

**Table 1:** Primer sequences used in this paper for qPCR.

Gene Name	Forward Sequence	Reverse Sequence
*18S*	CGCCGCTAGAGGTGAAATTCT	CATTCTTGGCAAATGCTTTCG
*Ppia*	TGTGCCAGGGTGGTGACTTTAC	TGGGAACCGTTTGTGTTTGG
*Ins1*	ACCCACCCAGGCTTTTG	CCCAGCTCCAGTTGTTCC
*Ins2*	ACCCACCCAGGCTTTTG	CCCAGCTCCAGTTGTTCC
*MafA*	CAGCGGCACATTCTGGA	CCCGCCAACTTCTCGTATT
*Pdx1*	CGTCCAGCTCCCTTTCC	ACGGGTCCTCTTGTTTTCC
*Ccnd1*	CAGAAGTGCGAAGAGGAGGTC	TCATCTTAGAGGCCACGAACAT
*Ccnd2*	TGTGGATTGTCTCAAAGCCTG	CAACATCCCGCACGTCTGTA
*Cdk1*	CTGCAGCTCGGAGCACAGTT	CCAGAACACGGAGGCACTTG
*Cdk4*	AGACCAGGACCTGAGGACAT	TCAGGTCCCGGTGAACAATG
*Chemerin*	GCCTGGCCTGCATTAAAATGG	CTTGCTTCAGAATTGGGCAGT
*Cmklr1*	GCCAACATACACGATGTCGC	GGATGTTGGGGTGTAGTGGG
*Gpr1*	TGAGCTCCTGCTACTTGTGC	AGGCAATGACCACAGACAGG
*Ccrl2*	GCCCCGGACGATGAATATGAT	CACCAAGATAAACACCGCCA

## Data Availability

Data are contained within the article or Supplementary Material.

## Supplementary Materials

The following supporting information can be downloaded at: https://www.mdpi.com/article/10.3390/ijms24119136/s1.
