# Supplementary material for: Chemerin as an Inducer of β Cell Proliferation Mediates Mitochondrial Homeostasis and Promotes β Cell Mass Expansion
Source: Int J Mol Sci. 2023 May 23;24(11):9136. doi: 10.3390/ijms24119136 (PMC10252465; doi:10.3390/ijms24119136)
Supplement: Supplementary file 1 [file ijms-24-09136-s001.zip › Supplementary Figures .pdf]

Supplementary Figures

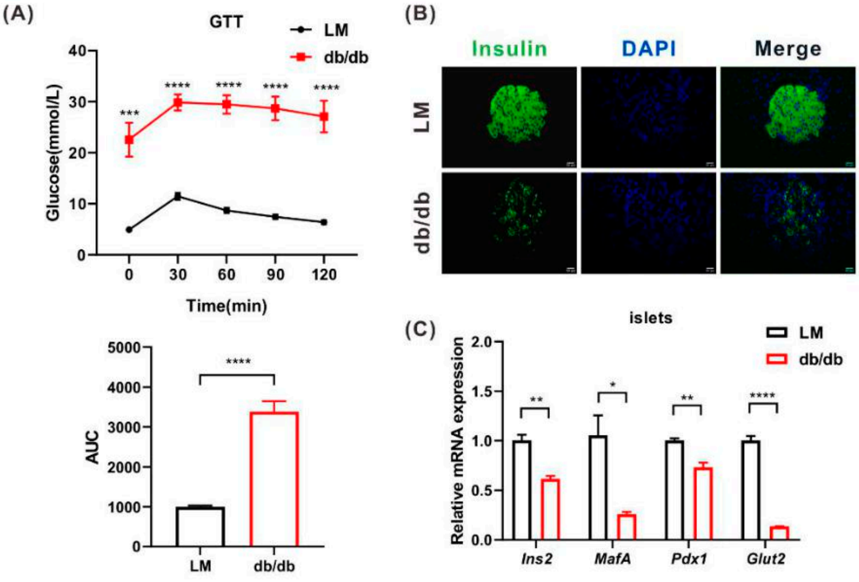

Figure S1. Islets function is impaired in *db/db* mice. **(A)** GTT assay in LM and *db/db* mice (n=8). **(B)** Immunofluorescence staining of pancreas tissues from LM and *db/db* mice (n=3). **(C)** qPCR analysis of *Ins2, MafA, Pdx1*, and *Glut2* genes expression in pancreatic islets in LM and *db/db* mice(n=3). * p <0.05; ** p <0.01; *** p <0.001; **** p <0.0001.

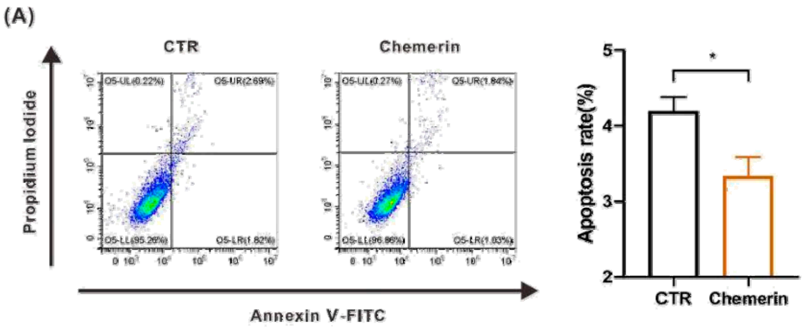

Figure S2. Analysis of apoptosis level in MIN6 cells treated with Chemerin analyzed by flow cytometry (n=3). * $p < 0.05$.

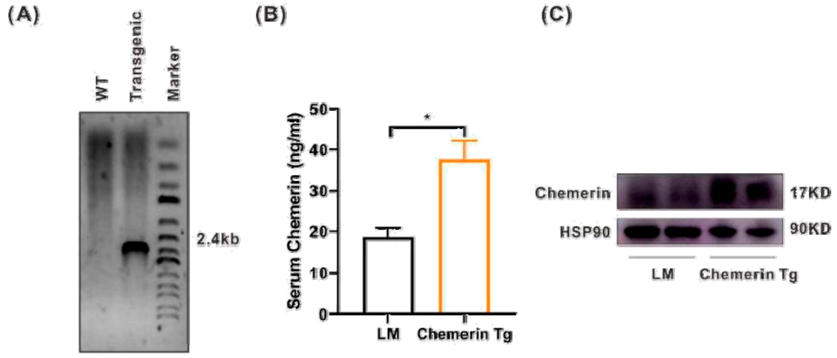

Figure S3. Identification of Chemerin Tg mice. **(A)** Identification of mice genotypes. **(B)** ELISA analysis of serum Chemerin in LM and Chemerin Tg mice. **(C)** Western blot analysis of Chemerin expression in white adipose tissue of LM and Chemerin Tg mice. * p <0.05

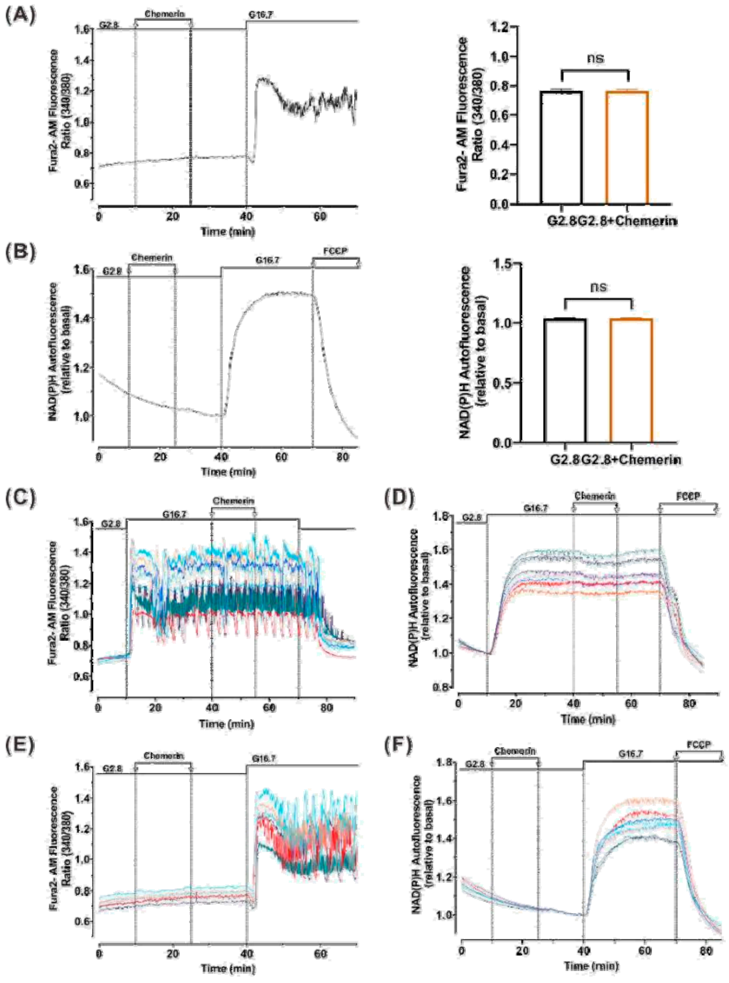

Figure S4. Effects of Chemerin on the cytosolic Ca^{2+} c and NAD(P)H fluorescence. Glucose increases Ca^{2+} c and NAD(P)H autofluorescence when glucose concentration was changed between G2.8 to G16.7. **(A, B)** At G2.8, adding Chemerin (200 ng/ml) did not affect Ca^{2+} c (A) (n=7 islets/ 3 mice) nor NAD(P)H fluorescence (B) (n=6 islets/ 3 mice). **(C, E)** Individual traces of Ca^{2+} c oscillation when adding Chemerin at G16.7 (C) (n=8) and G2.8 (E) (n=7). **(D, F)** Individual traces of NAD(P)H fluorescence when adding Chemerin at G16.7 (D) (n=8 islets/ 3 mice) and G2.8 (F) (n=9 islets/ 3 mice). ns, not significant.
