# Supplementary material for: Chemerin as an Inducer of β Cell Proliferation Mediates Mitochondrial Homeostasis and Promotes β Cell Mass Expansion
Source: Int J Mol Sci. 2023 May 23;24(11):9136. doi: 10.3390/ijms24119136 (PMC10252465; doi:10.3390/ijms24119136)
Supplement: Supplementary file 1 [file ijms-24-09136-s001.zip › Supplementary table S1.pdf]

Supplementary Table S1: Antibodies used in this study

Antibody	Company	Catalog number
HSP90	Proteintech	60318-1
Chemerin	Santa Cruz Technology	sc-373797
CMKLR1	Abcepta	AP50620
CCRL2	Thermo Fisher	PA5-75492
Insulin	Thermo Fisher	53-9769-82
BIK	Thermo Fisher	PA5-20249
Insulin	Cell Signaling Technology	8138S
Insulin	Cell Signaling Technology	4590S
MafA	Cell Signaling Technology	79737S
Cyclin D2	Cell Signaling Technology	3741T
PDX1	Proteintech	20989-1-AP
